# Proteomics-Based Characterization of miR-574-5p Decoy to CUGBP1 Suggests Specificity for mPGES-1 Regulation in Human Lung Cancer Cells

**DOI:** 10.3389/fphar.2020.00196

**Published:** 2020-03-13

**Authors:** Anne C. Emmerich, Julia Wellstein, Elena Ossipova, Isabell Baumann, Johan Lengqvist, Kim Kultima, Per-Johan Jakobsson, Dieter Steinhilber, Meike J. Saul

**Affiliations:** ^1^Department of Biology, Technische Universität Darmstadt, Darmstadt, Germany; ^2^Institute of Pharmaceutical Chemistry, Goethe-Universität Frankfurt, Frankfurt, Germany; ^3^Rheumatology Unit, Department of Medicine, Karolinska Institutet, Karolinska University Hospital, Stockholm, Sweden; ^4^Department of Medical Sciences, Clinical Chemistry, Uppsala University, Uppsala, Sweden

**Keywords:** miR-574-5p, CUGBP1, non-canonical miR function, decoy, proteomics, mPGES-1, lung cancer

## Abstract

MicroRNAs (miRs) are one of the most important post-transcriptional repressors of gene expression. However, miR-574-5p has recently been shown to positively regulate the expression of microsomal prostaglandin E-synthase-1 (mPGES-1), a key enzyme in the prostaglandin E_2_ (PGE_2_) biosynthesis, by acting as decoy to the RNA-binding protein CUG-RNA binding protein 1 (CUGBP1) in human lung cancer. miR-574-5p exhibits oncogenic properties and promotes lung tumor growth *in vivo* via induction of mPGES-1-derived PGE_2_ synthesis. In a mass spectrometry-based proteomics study, we now attempted to characterize this decoy mechanism in A549 lung cancer cells at a cellular level. Besides the identification of novel CUGBP1 targets, we identified that the interaction between miR-574-5p and CUGBP1 specifically regulates mPGES-1 expression. This is supported by the fact that CUGBP1 and miR-574-5p are located in the nucleus, where CUGBP1 regulates alternative splicing. Further, in a bioinformatical approach we showed that the decoy-dependent mPGES-1 splicing pattern is unique. The specificity of miR-574-5p/CUGBP1 regulation on mPGES-1 expression supports the therapeutic strategy of pharmacological inhibition of PGE_2_ formation, which may provide significant therapeutic value for NSCLC patients with high miR-574-5p levels.

## Introduction

MicroRNAs (miRs) represent a large family of small non-coding RNAs of approximately 21 nucleotide length. MiRs have proven to be important post-transcriptional regulators of gene expression in eukaryotes (Jansson and Lund, 2012). It is predicted that miRs control the expression of about 60% of human protein-coding genes (Friedman et al., 2009; Schwanhäusser et al., 2011) and participate in the regulation of almost all cellular processes. Importantly, they have emerged as critical regulators of tumorigenesis and progression of different types of cancer, since certain miRs can lead to a dysregulation of processes like cell differentiation (Harfe, 2005), cell cycle (Carleton et al., 2007), and apoptosis (Jovanovic and Hengartner, 2006). Mechanistically, miRs are known to repress expression of their target genes by base-pairing to 3′ untranslated regions (UTRs) (Wienholds and Plasterk, 2005). Depending on the level of complementarity, miR binding leads to either translational repression or degradation of the mRNA. Both regulatory miR functions result in strongly impaired gene expression and a decreased protein level (Guo et al., 2010).

In contrast to this, over the last years different studies have discovered new non-canonical miR functions. MiRs are not only able to inhibit gene expression, but also to activate gene expression. They can bind to RNA-binding proteins (RBPs) and sequester them from their target mRNA. This function is independent of the miR’s seed region and operates exclusively by interference with a RBP. It was shown for the first time for miR-328 which acts as RNA decoy for the heterogeneous nuclear ribonucleoprotein E2 (hnRNP E2), a global gene expression repressor (Eiring et al., 2010; Saul et al., 2016, 2019b).

Recently, a new miR/RBP interaction was discovered. MiR-574-5p reveals a binding site for CUG-RNA binding protein 1 (CUGBP1) in its mature form (Saul et al., 2019a). Therefore, it can directly interact with CUGBP1 and promotes lung tumor growth *in vivo* by inducing microsomal prostaglandin E-synthase-1 (mPGES-1) expression, the terminal synthase responsible for the production of the pro-tumorigenic lipid mediator prostaglandin E_2_ (PGE_2_) (Nakanishi and Rosenberg, 2013). In human lung tumor and under inflammatory circumstances, miR-574-5p is strongly upregulated and increases mPGES-1 expression by preventing CUGBP1 binding to the mPGES-1 3′UTR which leads to an enhanced alternative splicing and the generation of a novel 3′UTR isoform (Saul et al., 2019a). The newly discovered link between miR-574-5p overexpression and PGE_2_-mediated tumor growth *in vivo* suggests that pharmacological inhibition of PGE_2_ formation might be a potential therapeutic approach in combination with standard therapies for lung cancer patients with high miR-574-5p levels (Saul et al., 2019a). Thereby, miR-574-5p could serve as a stratification and biomarker to identify suitable candidates, an approach which is also supported by earlier studies (Foss et al., 2011; Del Vescovo et al., 2014).

To identify new CUGBP1 targets and characterize the global impact of the novel RNA decoy mechanism of miR-574-5p and CUGBP1, with the aim of demonstrating the specificity of the biomarker miR-574-5p, we applied a tandem mass tag (TMT)-based proteomics approach in A549 lung cancer cells. In this study, we were able to identify and validate novel CUGBP1 targets like Mothers against decapentaplegic homolog (SMAD) 2 which is an important signal transducer and transcription factor. Moreover, we demonstrate that the miR-574-5p/CUGBP1 decoy mechanism could be specific for mPGES-1 in human lung cancer due to a very unique splicing pattern in the 3′UTR of mPGES-1.

## Materials and Methods

### Cell Line and Cell Culture Conditions

The human lung adenocarcinoma cell line A549 (ATCC) was cultured in Dulbecco’s modified Eagle medium (DMEM, Life Technologies, Thermo Fisher Scientific) with 10% (v/v) fetal bovine serum (FBS, Life Technologies, Thermo Fisher Scientific), 100 U/ml penicillin, 100 μg/ml streptomycin and 1 mM sodium pyruvate (PAA the Cell Culture Company). Cell culture was carried out in a humidified atmosphere of 5% CO_2_ at 37°C.

### RNA Interference

A549 cells were seeded at a density of 5 × 10^5^ cells/well in a 6-well plate and treated with 20 pmol siRNA oligonucleotides the next day using Lipofectamine 2000^®^ (Invitrogen) according to manufacturer’s instructions. A previously published anti-CUGBP1 siRNA (5′-GCUGUUUAUUGGUAUGAUU-3′) was used for transient knockdown (Δ) of CUGBP1 (Vlasova-St Louis and Bohjanen, 2011). As control, a scramble siRNA with an unspecific sequence (5′-UCUCUCACAACGGGCAUUU-3′) was used. 24 h after transfection, A549 cells were incubated with 5 ng/ml of Interleukin (IL)-1β (Sigma-Aldrich) for further 24 h. The efficiency of the knockdown was determined by Western blot analysis as described in Saul et al. (2019a).

### Transfection of miR-574-5p Mimic or Inhibitor (miR Overexpression or miR Knockdown)

A549 cells were seeded at a density of 5 × 10^5^ per well in a 6-well plate. For overexpression of miR-574-5p, 100 pmol miRIDIAN miR-574-5p mimics (HMI0794, Sigma-Aldrich) or negative control mimic (HMC0002, Sigma-Aldrich) were transfected using Lipofectamine 2000^®^ according to the manufacturer’s instructions. In the same way, the A549 cells were transfected with 200 pmol miR-574-5p-LNA^TM^ inhibitor (4101451-001, Exiqon) or negative control LNA (199006-001, Exiqon) for the knockdown of miR-574-5p. 24 h after transfection, A549 cells were incubated with 5 ng/ml of IL-1β (Sigma-Aldrich) for further 24 h. The transfection efficiency was determined by qRT-PCR.

### Fraction Preparation

In order to find compartment specific regulations, soluble and microsomal proteins of A549 cells were separately analyzed. The fractionation was performed as previously described (Saul et al., 2016). Protein content of Western blot samples was determined by Bradford assay (Bio-Rad Laboratories), for proteomics samples the protein amount was determined by Pierce BCA Protein Assay (Thermo Fisher Scientific) following manufacturing instructions.

### Trypsin Digestion, TMT Labeling and Peptide Fractionation

The protein pellets were solubilized in 50 μl buffer containing 0.05 M triethylammonium bicarbonate, 4 M urea, 0.01% SDS and 2% RapiGest SF Surfactant (Waters). Equal protein amount from each sample (50 μg) was taken as starting point for further sample preparation. Disulfide reduction was performed by adding 5 μl 1 M DTT (in H_2_O) for 30 min at 56°C followed by sulfhydryl alkylation performed by adding 4 μl iodoacetamide solution (1 M in H_2_O) and incubation at RT for 1 h in the dark. Trypsin (modified sequencing grade, Promega) was added in a ratio of 1:30 (trypsin: protein) and the samples were incubated at 37°C overnight. Peptide labeling was performed using tandem mass tags (TMT 6-plex, Thermo Fisher Scientific) according to the instructions of the manufacturer (Table 1). Labeled peptide samples were pooled to final soluble and microsomal fractions and excess reagents were removed by solid phase extraction (STRATA XC Phenomenex). Liquid chromatography tandem MS of a TMT-labeled sample was performed on QExactive mass spectrometer (Thermo Fisher Scientific).

**TABLE 1:** TMT labeling of protein samples in the proteomics study.

Sample name	Fraction	Label reagent
Control ΔCUGBP1 (Scramble)	Soluble	126
ΔCUGBP1	Soluble	127
Control miR-574-5p oe	Soluble	128
miR-574-5p oe	Soluble	129
Control ΔmiR-574-5p	Soluble	130
ΔmiR-574-5p	Soluble	131
Control ΔCUGBP1 (Scramble)	Microsomal	126
ΔCUGBP1	Microsomal	127
Control miR-574-5p oe	Microsomal	128
miR-574-5p oe	Microsomal	129
Control ΔmiR-574-5p	Microsomal	130
ΔmiR-574-5p	Microsomal	131

Peptide pre-fractionation was done essentially as described (Cao et al., 2012). Briefly, TMT-labeled protein digests were separated over a 60 min gradient (3–55% B-buffer) on a 2.1 × 250 mm XBridge BEH300 C18 column (Waters) using a flow rate of 200 μL/min. A- and B-buffers consisted of 20 mM ammonia in MilliQ-grade water or 20 mM ammonia in 80% acetonitrile, respectively. Individual fractions were collected per minute and the fractions covering the peptide elution range were concatenated to yield 12 final pooled fractions. These fractions were evaporated to dryness by vaccume drying and stored frozen until nanoLC-MS data capture.

### Mass Spectrometry

Online LC-MS was performed using a Q-Exactive mass spectrometer (Thermo Scientific). Peptide samples were trapped on an Acclaim PepMap trap column (C18, 3 μm, 100Å, 75 μm × 20 mm), and separated on a 15-cm long C18 picofrit column (100 μm internal diameter, 5 μm bead size, Nikkyo Technos, Tokyo, Japan) installed on to the nano-electrospray ionization source. Solvent A was 97% water, 3% acetonitrile, 0.1% formic acid; and solvent B was 5% water, 95% acetonitrile, 0.1% formic acid. At a constant flow of 0.25 μl/min, the curved gradient went from 3% B up to 48% B in 50 min.

FTMS master scans with 70,000 resolution (and mass range 400–1200 m/z) were followed by data-dependent MS/MS (17,500 resolution) on the top 10 precursor ions using higher energy collision dissociation (HCD) at 31% normalized collision energy. Precursors were isolated with a 2 m/z window. Automatic gain control (AGC) targets were 3e6 for MS1 and 2e5 for MS2. Maximum injection times were 250 ms for MS1 and 200 ms for MS2. Dynamic exclusion was used with 20 s duration. Precursors with unassigned charge state or charge state 1 were excluded. An underfill ratio of 1% was used. The mass spectrometry proteomics data have been deposited to the ProteomeXchange Consortium via the PRIDE (Perez-Riverol et al., 2019) partner repository with the dataset identifier PXD016803.

### Data Analysis

Acquired MS raw files were searched using Sequest-Percolator under the software platform Proteome Discoverer 1.4.1.14 (Thermo Fisher Scientific) against human Uniprot database (release 01.12.2015) and filtered to a 1% FDR cut off. We used a precursor ion mass tolerance of 10 ppm, and product ion mass tolerances of 0.02 Da for HCD-FTMS. The algorithm considered tryptic peptides with maximum 2 missed cleavages; carbamidomethylation (C), TMT 6-plex (K, N-term) as fixed modifications and oxidation (M) as dynamic modifications. Quantification of reporter ions was done by Proteome Discoverer on HCD-FTMS tandem mass spectra using an integration window tolerance of 10 ppm. Only unique peptides in the data set were used for quantification. Fold values were calculated comparing proteins from ΔCUGBP1 to Scramble, ΔmiR-574-5p to negative control LNA and miR-574-5p oe to negative control mimic. Fold values of +1.5/−1.5 were considered up-/downregulated. All regulated proteins in soluble as well as microsomal fractions from all three conditions were analyzed using Ingenuity Pathway Analysis (IPA) (Ingenuity Systems^1^). We predicted the five most affected canonical pathways. The canonical pathways with *p*-values ≤ 0.05 were defined as significant.

### RNA Extraction

Total RNA was extracted with TRIzol reagent (Invitrogen) and treated with Turbo DNase (Ambion, Thermo Fisher Scientific) according to manufacturer’s instructions. RNA concentration was determined with NanoDrop (Peqlab). DNase-treated RNA was used for reverse transcription. For mRNAs, the High-Capacity cDNA reverse transcription Kit (Applied Biosystems, Thermo Fisher Scientific) was used according to manufacturer’s instructions. For miR detection, RNA was transcribed with the miScript II RT Kit (Qiagen) according to manufacturer’s instructions.

### Quantitative Reverse Transcription PCR (qRT-PCR)

qRT-PCR was performed with Applied Biosystems StepOne Plus^TM^ Real-Time PCR System (Applied Biosystem, Thermo Fisher Scientific). 1 μl cDNA (1:2 diluted) was used per reaction. For mRNA quantification, qRT-PCR was performed using Fast SYBR Green PCR Master Mix (Applied Biosystems, Thermo Fisher Scientific). The sequences for primer pairs are listed in Table 2. qRT-PCR based miR quantification was performed using miScript system (Qiagen). It was performed using the miR-574-5p specific primer (MS00043617, Qiagen). qRT-PCR was performed according to the manufacturer’s instructions. Fold inductions were calculated using 2(−ΔCt) value.

**TABLE 2:** Primers used for qRT-PCR analysis.

Target	Sequence
cJun fw	TCGACATGGAGTCCCAGGA
cJun rev	GGCGATTCTCTCCAGCTTCC
SMAD2 fw	GGGATGCTTCAGGTAGGACA
SMAD2 rev	TCTCTTTGCCAGGAATGCTT
SMAD3 fw	CGCAGAACGTCAACACCAAG
SMAD3 rev	GGCGGCAGTAGATGACATGA
COX-2 fw	CCGGGTACAATCGCACTTAT
COX-2 rev	GGCGCTCAGCCATACAG
NDUFS2 fw	GTTTTGCCCATCTGGCTGGT
NDUFS2 rev	CATGCCATGGCCTATGGTGAA
mPGES-1 fw	GAAGAAGGCCTTTGCCAAC
mPGES-1 rev	CCAGGAAAAGGAAGGGGTAG
UBE2R2 fw	ATGTGGCACCCCAACATT
UBE2R2 rev	TCCACCTTTCAGAAGGCAGT
CEP41 fw	ACAGAACCCAAGATACCAGCATAT
CEP41 rev	GGGAGCTGGTAAGATACACACA
SLC39A6 fw	GCACTTACTGCTGGCTTATTCA
SLC39A6 rev	CGGCTACATCCATGGTCACT
PAIP2 fw	CCATTTGCAGAGTACATGTGGA
PAIP2 rev	CCGTACTTCACCCCAGGAAC
GTF2E2 fw	CCATGCAGGAATCTGGACCA
GTF2E2 rev	AATCCTTCAGCACTCCAGCC
LEO1 fw	ACTGCCCAACTTTCTCAGTGT
LEO1 rev	AGATGATTGTGGTCGCCCTG

### RNA Immunoprecipitation (RIP)

RNA immunoprecipitation was performed as previously described (Saul et al., 2019a). In short, 6 × 10^6^ A549 cells per RIP were resuspended in lysis buffer containing 10 mM Tris-HCl (Carl Roth) pH 7.5, 10 mM KCl (Sigma-Aldrich), 1.5 mM MgCl_2_, 0.5 mM DTT (Sigma-Aldrich), 0.9% Nonidet P-40 (Sigma-Aldrich), 20 μl ribonuclease inhibitor and protease inhibitor EDTA-free (Roche). The suspension was sonicated. Afterward, samples were centrifuged, supernatant was transferred into a fresh tube and 10% were taken as input sample. We used blocked GammaBind Plus Sepharose beads (GE Healthcare). Beads and antibodies were linked by mixing 50 μl bead suspension with 10 μg of CUGBP1 antibody (05-621 clone3B1, Merck) or normal mouse IgG antibody (12-371, Merck). The IP was then conducted by dividing the lysate equally to the CUGBP1-/IgG-bead mixture and incubating for 2 h at 4°C. Afterward, samples were washed and 10% of each precipitate was taken for Western blot analysis to validate the IP. The remaining precipitates were resuspended in 500 μl TRIzol reagent (Invitrogen) and RNA was isolated as described above. Subsequent qRT-PCR based mRNA or miR quantification was performed as previously described. All buffers and solutions contained protease inhibitor EDTA-free (Roche). Successful precipitation was verified via Western blot analysis with a primary antibody directed against CUGBP1 (ab129115, Abcam).

### Western Blotting

Western blot analysis was performed as previously described (Ochs et al., 2013). Briefly, the Odyssey Imaging System (Li-COR Biosciences) was used which allows a linear quantification using near-infrared fluorescence. The membranes were incubated with primary antibodies that recognize CUGBP1 (ab9549, Abcam), SMAD2 (sc-6200, Santa Cruz), SMAD3 (ab28379, Abcam), SMAD4 (ab3219, Abcam), p38 (sc-535, Santa Cruz), NDUFS2 (ab96160, Abcam), mPGES-1 [160140, Cayman-Chemical (Westman et al., 2004)], β-Actin (sc-1616, Santa Cruz).

### Bioinformatical 3′UTR Analysis

For the analysis of splice patterns, we concentrated on the list of proteins that were upregulated at least 1.5-fold in response to ΔCUGBP1 concerning the mass spectrometry data. All described 3′UTR sequences of those 399 proteins were downloaded from ensemble biomart [GRCh38/.p12, version 91, Ensembl variation resources (Hunt et al., 2018)], resulting in a list of 1916 transcripts. Those were aligned with 42 different binding motifs of CUGBP1 that were known so far [downloaded from the online tool Splice Aid F (Marquis et al., 2006; Giulietti et al., 2013)].

Then, three criteria were applied (high stringency analysis): (I) binding sites should be of 39 or 46 nucleotides (nt) length, (II) there should be 2 binding sites and (III) those binding sites should span a potential intron of 1000 nt. For a second less stringent approach (referred to as low stringency analysis), we investigated transcripts (I) with binding sites of at least 8 nt length (II), at least 2 binding sites and (III) those binding sites should span a potential intron of 100 nt. Analysis was conducted using Microsoft Excel.

### Immunofluorescence

Immunofluorescent staining was performed as previously described (Saul et al., 2019a). A549 cells were seeded on glass cover slips (12 mm, Neolab) at a density of 2.5 × 10^5^ per well in a 6-well plate and incubated for 24 h. Cells were washed with PBS, prior to fixation with 4% formaldehyde (FA, Carl Roth) for 10 min. After washing with PBS 3 times for 3 min, cells were permeabilized with 0.5% Triton X-100 (Sigma-Aldrich) in PBS for 10 min. Then, cells were blocked with 2% BSA (Sigma-Aldrich) in PBS for 20 min. The primary antibody directed against CUGBP1 (ab9549, Abcam) was diluted in blocking solution (1:500) and incubated for 1 h at room temperature together with fixed cells. Afterward, cells were washed 3 times with 0.01% Tween20 (Carl Roth) in PBS for 5 min and incubated for 45 min at room temperature with the secondary antibody goat anti-mouse IgG (Alexa Fluor^®^ 594, ab150116, Abcam) diluted in blocking solution (1:500). Finally, cells were washed with 0.01% Tween20 (Carl Roth) in PBS as described, counterstained for 5 min with 4′,6-diamidine-2′-phenylindole dihydrochloride (DAPI, Sigma-Aldrich) and mounted in Mowiol 4-88 mounting medium (Sigma-Aldrich).

### Fluorescent *In situ* Hybridization (FISH)

FISH was performed as previously described (Saul et al., 2019a). 2.5 × 10^5^ A549 cells were seeded on glass cover slips (12 mm, Neolab) in 6-well plates and incubated for 24 h. Cells were washed with PBS and prefixed with 1% FA in PBS for 10 min. After 3 washing steps of 3 min with PBS, cells were permeabilized with 0.5% Triton X-100 in PBS for 20 min on ice. After additional three washing steps of 3 min with PBS, cells were refixed with 4% FA for 10 min followed by further washing with PBS 3 times for 3 min. Subsequently, samples were prehybridized for 30 min at 40°C in microRNA ISH buffer (Qiagen) and then hybridized for 1 h at 54°C with 100 nM double digoxigenin (DIG) labeled miR-574-5p probe (Qiagen) diluted in microRNA ISH buffer. After hybridization, samples were washed twice for 5 min with 2x saline-sodium citrate buffer (Gibco, Carlsbad, CA, United States) at hybridization temperature and once at room temperature followed by a 20 min blocking step with 2% BSA in PBS. Subsequently, cells were incubated for 1 h at room temperature with rabbit anti-DIG antibody (9H27L19, Invitrogen, Thermo Fisher Scientific) diluted 1:40 in blocking solution. Afterward, cells were washed with 0.01% Tween20 in PBS 3 times for 5 min and incubated at room temperature for 45 min with secondary antibody goat anti-rabbit IgG (Alexa Fluor^®^ 594, 111-585-144, Jackson ImmunoResearch) diluted 1:300 in blocking solution. Then, cells were washed with 0.01% Tween20 in PBS, counterstained with DAPI and mounted as described.

### Microscopy

The Leica TCS SPE confocal point scanner mounted on a Leica DMi8 stand equipped with an oil immersion 63 × Apochromat was used in order to take confocal images of immunofluorescence and miR FISH samples. For all samples, the 405 and 561 nm laser lines were used to perform excitation. All images were analyzed using the ImageJ software and show one focal plane of the middle of the nucleus.

### Statistics

Results are shown as mean + Standard error of mean (SEM) of at least three independent experiments. Statistical analysis was carried out by Student’s paired or unpaired *t*-test (two-tailed) using GraphPad Prism 5.0. Differences were considered as significant for *p* ≤ 0.05 (indicated as ^∗^ for *p* ≤ 0.05, ^∗∗^ for *p* ≤ 0.005, and ^∗∗∗^ for *p* ≤ 0.001).

## Results

### Analysis of Protein Expression Changes in Response to ΔCUGBP1, ΔmiR-574-5p, and miR-574-5p oe in IL-1β-Stimulated A549 Cells

**FIGURE 1:**
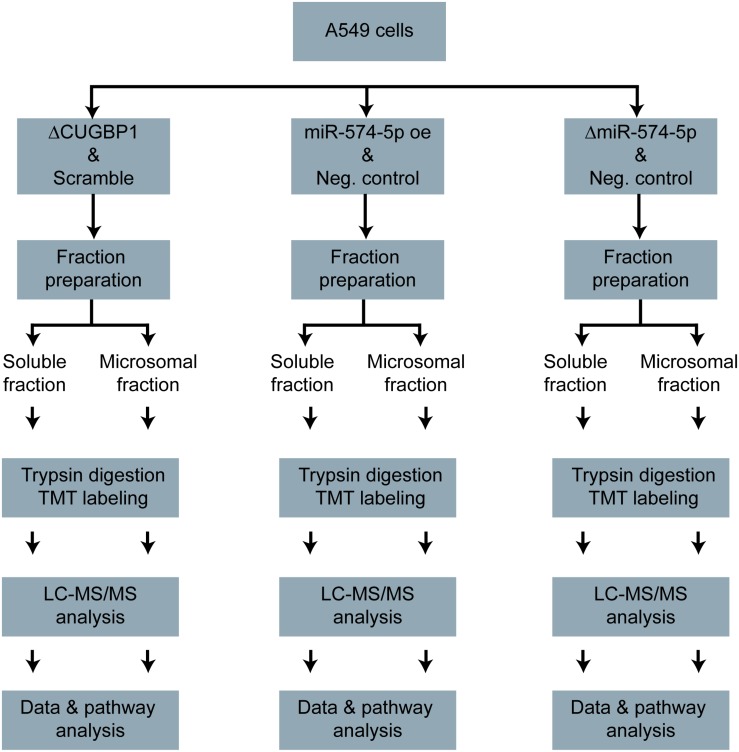
Proteomics study workflow. A549 cells with ΔCUGBP1, ΔmiR-574-5p, or miR-574-5p oe and respective controls. Soluble and microsomal proteins were isolated, trypsin digested and TMT-labeled. Afterward, the LC-MS/MS measurement was conducted, the data were analyzed and the pathway analysis was performed. Δ, knockdown; oe, overexpression; TMT, tandem mass tag.

**FIGURE 2:**
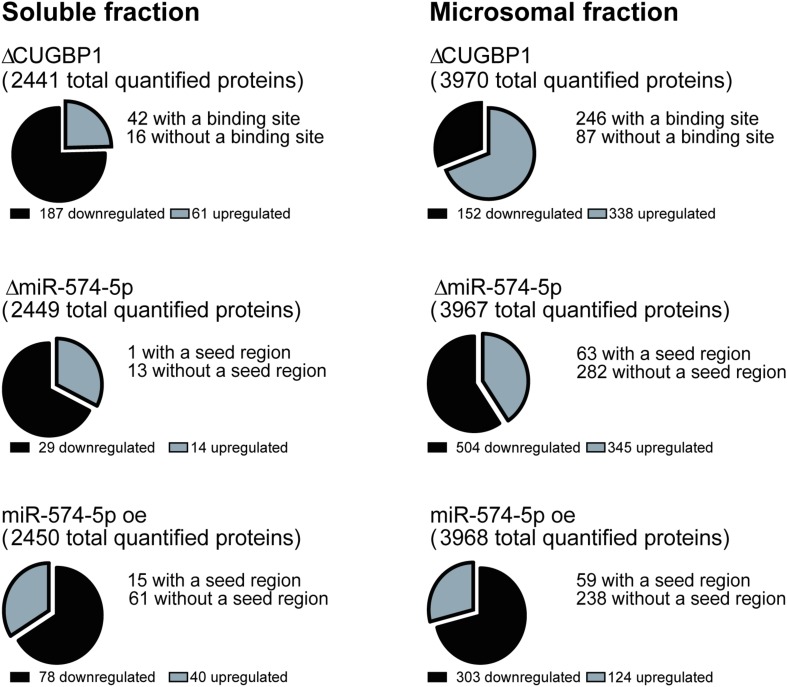
Numbers of regulated proteins. Numbers of differentially expressed proteins upon ΔCUGBP1, ΔmiR-574-5p, or miR-574-5p oe in soluble and microsomal fraction of the proteomics study. Cut-offs were set to 1.5-fold for upregulation and -1.5-fold for downregulation. CUGBP1 binding sites of upregulated proteins were predicted using splice aid www.introni.it/spliceaid.html, while miR-574-5p seed regions were identified with the online tool microrna.org. of proteins revealing an upregulation upon ΔmiR-574-5p or a downregulation upon miR-574-5p oe. Δ, knockdown; oe, overexpression.

A stable isotope labeling based proteomics study was conducted to identify changes in the proteome of A549 lung cancer cells stimulated with IL-1β (Figure 1). A549 cells with ΔCUGBP1, ΔmiR-574-5p or miR-574-5p oe were compared to their corresponding controls. Knockdown of CUGBP1 was validated via Western blot analysis and showed a reduction of 67% in the soluble fraction and 83% in the microsomal fraction of A549 cells (Supplementary Figure S1). Knockdown and overexpression of miR-574-5p were quantified with qRT-PCR analysis and revealed a significant ∼80% decrease of miR-574-5p, while miR-574-5p was ∼300-fold upregulated in the miR-574-5p oe samples (Supplementary Figure S1). For the LC-MS/MS analysis, all samples were digested and were labeled with TMT 6-plex to allow quantitative protein comparison (Table 1). In all three conditions (ΔCUGBP1, ΔmiR-574-5p or miR-574-5p oe) we identified roughly the same numbers of total protein (in soluble fraction around 2450 and in microsomal fraction around 3970 proteins). But only small percentages of them revealed an up- or downregulation in comparison to their corresponding controls (Figure 2). Overall, we set the criteria for upregulation to a TMT ratio of ≥1.5 (fold change ≥ 1.5) and downregulation was considered with a TMT ratio of ≤0.5 (fold change ≤ −1.5). In ΔCUGBP1 cells, canonical targets of CUGBP1 as well as decoy targets were supposed to show an upregulation in response to the knockdown. In the soluble fraction, 2% were indeed upregulated (61 proteins) and 8% (187 proteins) of all detected proteins were downregulated. In the microsomal fraction, 9% (338 proteins) were elevated, while 4% (152 proteins) showed a downregulation. In miR-574-5p oe cells, we expected direct miR targets to be decreased and decoy targets to be increased in response to high miR-574-5p levels. In the soluble fraction, 2% (40 proteins) were up- and 3% (78 proteins) were downregulated. While a decrease was measured for 8% of microsomal proteins (303 proteins), only 3% were upregulated (124 proteins). In the soluble fraction, 1% (29 proteins) of the total protein amount was decreased upon ΔmiR-574-5p, while 14 proteins showed an increase. However, the microsomal proteins showed higher percentages, 9% were upregulated (345 proteins), while even 13% (504 proteins) showed a downregulation.

Overall, the results show that on average 11% of all analyzed proteins are regulated by CUGBP1, while 9.7% of all detected proteins show an expression change related to miR-574-5p. But the distribution of the miR-574-5p regulated proteins varies considerably depending on the protein fraction.

### IPA Predicts Top Canonical Pathways and Upstream Regulators

Next, we analyzed all regulated proteins in soluble as well as microsomal fraction using IPA (Ingenuity Systems). The most affected canonical pathways as well as upstream regulators were predicted. Canonical pathways with *p*-values ≤ 0.05 were termed as significant. The analysis revealed that the Eukaryotic Initiation Factor 2 (eIF2) Signaling, Regulation of Eukaryotic Initiation Factor 4 (eIF4) and 70 kDa ribosomal S6 kinase (p70S6K) Signaling, tRNA Charging, Protein Ubiquitination Pathway and mechanistic Target of Rapamycin (mTOR) Signaling are the most affected canonical pathways in the soluble fraction (Supplementary Table S4). In the microsomal fraction, top regulated pathways were eIF2 Signaling, Protein Ubiquitination Pathway, Mitochondrial Dysfunction, Regulation of eIF4 and p70S6K Signaling as well as Oxidative Phosphorylation. Top predicted upstream regulators were Hepatocyte Nuclear Factor 4 Alpha (HNF4A), Cystatin D (CST5), Rapamycin-Insensitive Companion of mTOR (RICTOR), MYC Proto-Oncogene, MYCN Proto-Oncogene, microtubule associated protein tau (MAPT) and Tumor Protein P53 (TP53) (Supplementary Table S3), which further underlines the cancer context of CUGBP1 and miR-574-5p.

### Validation of TMT Based Quantification of Proteomics Results Using Western Blot Analysis

In order to validate the results of the proteomics study (Supplementary Tables S1, S2), distinct proteins were selected to be confirmed via Western blot analysis. Since this was a complex study with many different conditions (Figure 1), we concentrated not necessarily on physiologically connected proteins. We firstly selected NADH-Ubiquinone Oxidoreductase Core Subunit S2 (NDUFS2) which is a postulated CUGBP1 target in HeLa cells (Rattenbacher et al., 2010). Although there were no strong changes of microsomal NDUFS2 in the proteomics study, soluble NDUFS2 showed a trend toward slight upregulation in response to ΔCUGBP1 and miR-574-5p oe. This upregulation and also the values for the microsomal fraction could be confirmed using Western blot analysis (Supplementary Figure S2A, right panel). In response to ΔmiR-574-5p, soluble NDUFS2 revealed a slight decrease in the proteomics data which was also confirmed by Western blot analysis (Supplementary Figure S2A, left panel).

In addition to this, SMAD3 was another target which we aimed to validate. It was strongly downregulated in response to ΔCUGBP1 in the soluble fraction of the proteomics study and we were also able to confirm this via Western blot (Supplementary Figure S2B, left panel). While the proteomics study revealed no strong regulation of SMAD3 in microsomal fraction, IPA predicted it to be activated upon ΔCUGBP1 (Supplementary Table S3). Indeed, we observed an upregulation of SMAD3 proteins in the microsomal fraction performing Western blot analysis (Supplementary Figure S2B, right panel). In this case, we could validate the pathway analysis which is based on the proteomics data (Supplementary Tables S1, S2). In response to ΔmiR-574-5p, SMAD3 was slightly upregulated in the microsomal fraction which was also a tendency in the proteomics study, although weaker. In general, the Western blot data of the two miR-574-5p conditions showed higher variations in SMAD3 levels.

SMAD2, another member of the same protein family, was also predicted in the IPA to be upregulated upon ΔCUGBP1 (Supplementary Table S3). Indeed, we were able to confirm this regulation by Western blot analysis. We observed a significant fourfold upregulation of SMAD2 in the microsomal fraction (Supplementary Figure S2C, right panel). Interestingly, in the soluble fraction SMAD2 expression was significantly reduced (Supplementary Figure S2C, left panel) which indicates that its regulation might be highly compartment specific. In soluble and microsomal fraction, SMAD2 and SMAD3 revealed a slight downregulation in response to miR-574-5p oe and an upregulation in response to ΔmiR-574-5p in the microsomal fraction. This indicated that both genes might be canonical miR-574-5p targets. Therefore, we analyzed the 3′UTRs of SMAD2 and SMAD3 using the online tool microrna.org (Betel et al., 2010) but found that none of them contains a seed region of miR-574-5p. This suggests that the effects on protein level are caused by secondary effects.

To complete the family of SMADs, it was coherent to analyze SMAD4, as the only other SMAD that was detected in the proteomics study. However, neither in the proteomics study nor in the Western Blot images, it did depict any regulation upon ΔCUGBP1 (Supplementary Figure S3A) which validated the mass spectrometry data, but excluded SMAD4 as interesting potential target.

Finally, we studied p38 (MAPK14) which was one of the strongest downregulated proteins upon ΔCUGBP1 in the soluble fraction of the proteomics study. In fact, we verified this severe decrease of p38 protein using Western blot analysis (Supplementary Figure S3B). Nevertheless, this effect seems to be secondary, as CUGBP1 targets are supposed to be upregulated in response to the knockdown.

Overall, these results validated the proteomics data (Supplementary Tables S1, S2). Since we measured an increase in NDUFS2, SMAD2 and SMAD3 as a response to ΔCUGBP1, we considered them as potential novel CUGBP1 targets.

### Identification of New CUGBP1 Targets

Next, we aimed to identify new targets of the decoy mechanism, but also CUGBP1 targets using bioinformatical approaches. Therefore, we analyzed proteins which were increased upon ΔCUGBP1 and analyzed their 3′UTRs for CUGBP1 binding sites. We downloaded the 3′UTR sequences of the specific proteins from the database UCSC Genome Browser (December 2013 GRCh38/hg38)^2^ (Kent et al., 2002) and analyzed them by using the online tool SpliceAid 2 (Piva et al., 2009) to identify potential CUGBP1 binding motifs. Thereby, the algorithm detects GU-rich elements (GREs) and other binding motives (e.g., CUGUCUG) in the provided 3′UTR sequences. In fact, a high number of the upregulated proteins does have a potential CUGBP1 binding site: in the soluble fraction 69% (42 proteins) and in the microsomal fraction 73% (246 proteins) of all detected proteins in the proteomics study (Figure 2).

**FIGURE 3:**
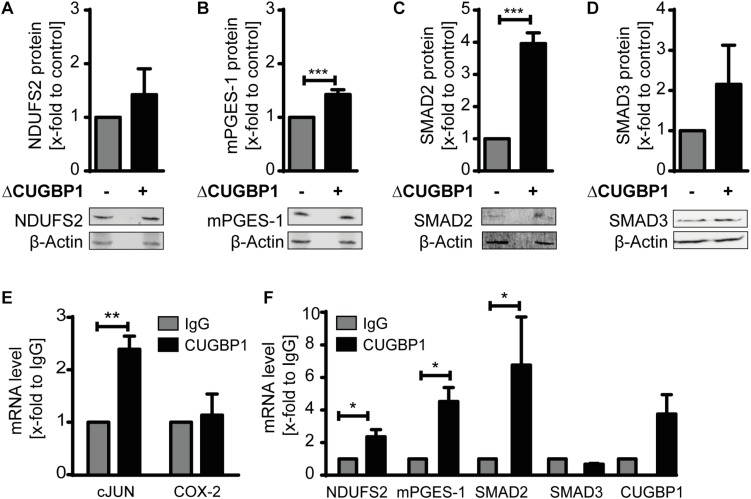
Identification of novel CUGBP1 targets. Western blots of IL-1β-stimulated A549 cells with ΔCUGBP1 showed increased levels of **(A)** NDUFS2, **(B)** mPGES-1, **(C)** SMAD2 and **(D)** SMAD3 compared to control. β-Actin was used as loading control. Fold induction is given as mean (+SEM) of three independent experiments, *t*-test *** *p* ≤ 0.001. **(E,F)** RIP with antibodies against CUGBP1 or IgG (mock) showed enrichment of bound mRNAs quantified via q RT-PCR. **(E)** cJUN was used as positive control, COX-2 as negative control. **(F)** mRNAs of NDUFS2, mPGES-1, SMAD2 and CUGBP1 were enriched in CUGBP1-IP, while SMAD3 mRNA showed no enrichment. Relative enrichment normalized to IgG is given as mean (+SEM) from three independent experiments. *t*-test * *p* ≤ 0.05; ** *p* ≤ 0.005. RIP: RNA immunoprecipitation.

Since CUGBP1 itself was mainly detectable in the microsomal fraction (Supplementary Figure S1A), we focused on this fraction for further analysis. As a positive control we used mPGES-1 which is already described as CUGBP1 target and decoy target (Saul et al., 2019a). The three candidates SMAD2, SMAD3 and NDUFS2 all depicted an upregulation in response to ΔCUGBP1 visualized by Western blot analysis (Figures 3A,C,D) and indeed also contained binding sites for CUGBP1 in their 3′UTRs. SMAD2 and SMAD3 both exhibit multiple binding motifs all over their 3′UTRs, while NDUFS2 with a rather short 3′UTR, harbors only one binding site.

We also observed an increase on protein level of mPGES-1 upon ΔCUGBP1 which further supports our results from the mass spectrometry (Figure 3B). This data provides first evidence that SMAD2, SMAD3 and NDUFS2 could be CUGBP1 targets in A549 cells.

### RIP Confirmed Binding of CUGBP1 to mRNAs of Novel Targets

In order to further validate the new postulated targets SMAD2, SMAD3, NDUFS2 as well as mPGES-1, binding of CUGBP1 needed to be confirmed. Therefore, we performed immunoprecipitation of CUGBP1 with a specific antibody and then quantified the bound mRNAs via qRT-PCR. As mock control we used a normal mouse IgG antibody. Binding was assumed, if the specific mRNA showed an enrichment in CUGBP1-IP, compared to IgG-IP. Successful RIP was verified using Jun Proto-Oncogene AP-1 Transcription Factor Subunit (cJUN) as positive control (Figure 3E) which revealed a significant 2.4-fold enrichment. Cyclooxygenase (COX)-2 was used as negative control, since it has no binding site and was not influenced by CUGBP1 (Saul et al., 2019a). Indeed, we could show that CUGBP1 binds to three of the mRNAs (Figure 3F): SMAD2 mRNA was significantly 6.4-fold enriched. NDUFS2 showed a 2.2-fold enrichment and mPGES-1 was 4.3-fold enriched in comparison to IgG control. However, SMAD3 was not bound by CUGBP1 and showed no enrichment of the mRNA although it contains a binding site in the 3′UTR. Interestingly, it was also revealed that CUGBP1 binds its own mRNA, as it showed a slight enrichment compared to IgG, indicating an autoregulatory mechanism.

Overall, we confirmed CUGBP1 binding to three of the four postulated targets: SMAD2, NDUFS2 and mPGES-1. Since we assumed decoy targets to be a subpopulation of CUGBP1 targets, we aimed to investigate if these new candidates were also regulated by miR-574-5p.

### Identification of Novel miR-574-5p/CUGBP1 Decoy Targets by Investigating a “Decoy Regulation Pattern” on Protein Level

In order to find new targets of the miR-574-5p/CUGBP1 decoy mechanism, we analyzed the proteomics data in regard to a “decoy regulation pattern” in the three conditions (ΔCUGBP1, ΔmiR-574-5p or miR-574-5p oe) and aimed to verify it via Western blot analysis. As subpopulation of CUGBP1 targets, decoy targets should be increased in response to ΔCUGBP. Further, they are supposed to be downregulated upon ΔmiR-574-5p, because lower miR levels cause higher binding capacity of CUGBP1 which in turn has a negative effect on the protein level. *Vice versa*, miR-574-5p oe should lead to an upregulation of potential decoy targets, as more miRs are available to prevent binding of CUGBP1. Here, we also included those proteins showing a tendency for the “decoy regulation pattern,” such as only two conditions that met the criteria or only slight up- or downregulations, for instance NDUFS2 showed only a −1.33-fold induction in response to ΔCUGBP1. Another example is High-mobility group AT-hook 2 (HMGA2) protein which was strongly upregulated upon ΔCUGBP1 and downregulated upon ΔmiR-574-5p but showed only a 1.14-fold TMT ratio in response to miR-574-5p oe. As mentioned above, we focused only on the microsomal fraction, since CUGBP1 was predominantly expressed there (Supplementary Figure S1). We analyzed the newly postulated CUGBP1 target proteins and several other candidates via Western blot. Although the control decoy target mPGES-1 could be successfully validated, none of the other potential candidates could be confirmed revealing the “decoy regulation pattern” in the Western blot images. Neither SMAD2, SMAD3 nor NDUFS2 showed the adequate “decoy regulation pattern” concerning the two miR conditions (Supplementary Figure S2). Other candidates such as glyoxalase domain-containing protein 4 (GLOD4) or HMGA2 which were detected by mass spectrometry, could not be validated herein. Probably, the sensitivity of the Western blot system was too low and the proteins were only expressed on a very basal level by the cells.

**FIGURE 4:**
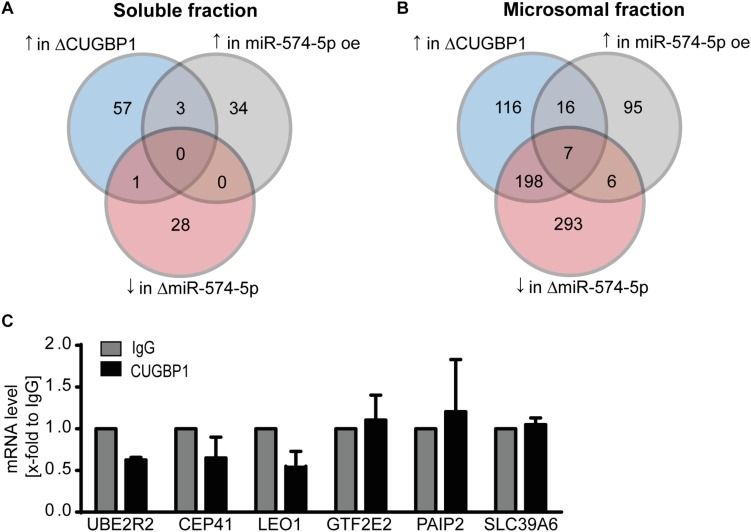
RIP excludes seven potential decoy candidates. Stringent criteria applied to results of the proteomics study: number of proteins which reveal an upregulation (↑) upon ΔCUGBP1, an upregulation upon miR-574-5p oe and a downregulation (↓) upon ΔmiR-574-5p in **(A)** soluble fraction and **(B)** microsomal fraction. **(C)** In RIP assays of A549 cells, none of the mRNAs was enriched in CUGBP1-IP samples compared to IgG. Relative enrichment normalized to IgG is given as mean (+SEM) from three independent experiments. Δ, knockdown; oe, overexpression; RIP, RNA immunoprecipitation.

We next analyzed the proteomics data again using more stringent criteria. Taking a closer look how many proteins showed a strong upregulation of ≥1.5-fold in response to ΔCUGBP1 and a downregulation of ≤−1.5-fold after ΔmiR-574-5p and at least an upregulation of ≥1.5-fold in response to miR-574-5p oe. With these criteria set, zero proteins were found in the soluble fraction (Figure 4A), while in the microsomal fraction only seven proteins (0.2%) matched this regulation pattern in all three conditions (Figure 4B). This gave us the first hint that the CUGBP1/miR-574-5p decoy seems to be a very specific mechanism, considering that originally around 3970 proteins were detected in the microsomal fraction in the proteomics study. Those seven proteins were: Ubiquitin Conjugating Enzyme E2 R2 (UBE2R2), Centrosomal Protein of 41 kDa (CEP41), RNA polymerase-associated protein LEO1 (LEO1), General Transcription Factor IIE Subunit 2 (GTF2E2), Polyadenylate-Binding Protein-Interacting Protein 2 (PAIP2), Solute carrier family 39 member 6 (SLC39A6), GRIP1 Associated Protein 1 (GRIPAP1). Of note, mPGES-1 was not among the seven proteins, since it did not reveal the exact pattern in the proteomics data either, probably due to the limitations of the mass spectrometry procedure. While GRIPAP1 has no described 3′UTR, the other 3′UTRs were analyzed concerning CUGBP1 binding motifs and it was revealed that all of them did contain a binding site in their 3′UTRs. By conducting a RIP binding assay, we analyzed whether the potential decoy candidates were bound by CUGBP1. It turned out that none of these six remaining mRNAs showed an enrichment in the CUGBP1-IP compared to mock control (Figure 4C). They were excluded as decoy targets in A549 cells, since none of the candidates was bound by CUGBP1. Thus, based on our Western blot and RIP data, mPGES-1 is the only target that was identified to be regulated by the decoy mechanism.

### Subcellular Localization of CUGBP1 and miR-574-5p in A549 Cells

**FIGURE 5:**
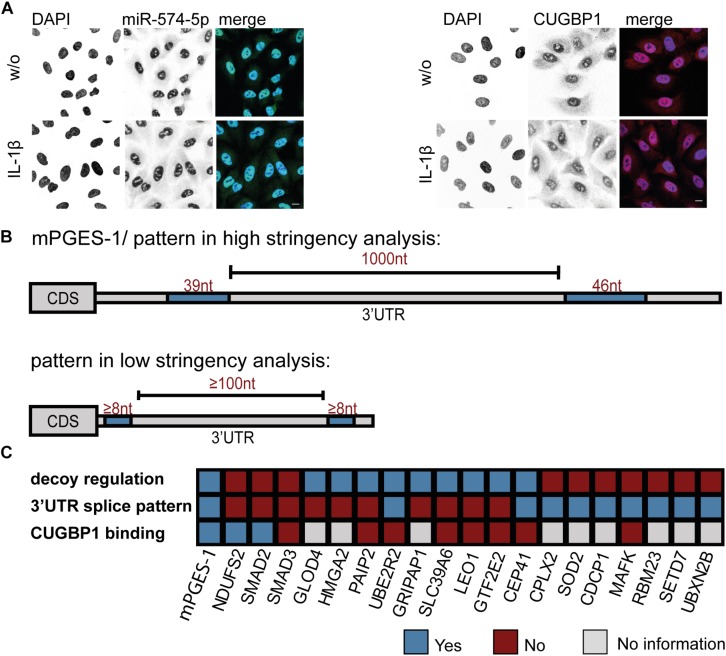
Bioinformatical 3′UTR analysis confirms specificity of mPGES-1-derived splicing pattern. **(A)** FISH assay and immunofluorescence staining of A549 cells ± IL-1β. miR-574-5p FISH was performed using DIG-labeled miRCURY LNA probes (green). Immunofluorescence staining with specific antibody visualized subcellular localization of CUGBP1 (red). Nuclei were counterstained with DAPI (blue). Scale bar represents 10 μm in each image. Representative images of three independent staining experiments are shown. **(B)** Bioinformatical 3′UTR analysis of upregulated proteins from proteomics study after ΔCUGBP1. High stringency/mPGES-1 splice pattern (upper panel) or low stringency pattern (lower panel) with two or more CUGBP1 binding sites (≥8 nucleotides), with potential intron (≥100 nucleotides). **(C)** Summary of the decoy target search. 20 different proteins were analyzed concerning their “decoy regulation pattern” on protein level, the specific (low stringency) splice pattern in the 3′UTR and binding of CUGBP1 to their mRNA in RIP assays. Nt, nucleotides; FISH, fluorescence *in situ* hybridization; RIP, RNA immunoprecipitation. Δ, knockdown.

In order to approach the decoy search on a different level, we wanted to find out exactly where in the cell the interaction of CUGBP1 and miR-574-5p takes place. Therefore, subcellular localization of the two binding partners was visualized using immunofluorescence staining and FISH assay, respectively. miR-574-5p was detected using a complementary DIG labeled LNA probe which was visualized with a DIG binding antibody. For localization of CUGBP1, immunofluorescence staining with a specific antibody directed against CUGBP1 was performed. DAPI served as nuclear marker. In fact, it was possible to show that both, miR-574-5p and CUGBP1 were mainly located within the nuclei of A549 cells (Figure 5A). While miR-574-5p showed very weak cytoplasmic signals, CUGBP1 showed a stronger presence in the cytoplasm, but was still predominantly located in the nucleus. The cellular localization of both binding partners did not change upon IL-1β stimulation. Therefore, we concluded that the decoy mechanism mainly takes place in the nucleus and miR-574-5p interferes with CUGBP1 functions there, just like it is the case for the mPGES-1 mRNA regulation.

### Bioinformatical 3′UTR Analysis Revealed That Splicing Pattern of mPGES-1 3′UTR Is Very Unique

Since both CUGBP1 and miR-574-5p were located in the nucleus, it was consequential to concentrate on alternative splicing which is the main function of CUGBP1 within the nucleus. In the case of mPGES-1, as the decoy target model, CUGBP1 binds to two binding sites in the mPGES-1 3′UTR and influences alternative splicing which creates a shorter 3′UTR isoform. Therefore, we used this as a model and analyzed other potential decoy candidates concerning this splicing pattern. Sequences of the 3′UTRs from all 399 proteins which showed an upregulation in response to ΔCUGBP1 in soluble or microsomal fraction of the proteomics study were downloaded, using ensemble biomart GRCh38/.p12, version 91, Ensembl variation resources (Hunt et al., 2018). These 1914 3’UTR sequences were aligned with 42 described CUGBP1 binding motifs [using the online tool Splice Aid F (Giulietti et al., 2013)]. In a high stringency analysis, we searched for the exact splicing pattern of mPGES-1, and found that none of the transcripts represented this exact pattern, with two 39 and 46 nt binding sites and 1000 nt in between (Figure 5B, upper panel). Therefore, it was also considered that the splicing pattern might not have to be identical which is why we loosened the criteria in a second low stringency analysis. We searched for transcripts containing two CUGBP1 binding sites of at least 8 nt length and with at least 100 nt in between (Figure 5B, lower panel). As depicted in Table 3, 575 (30%) of the originally 1914 3′UTRs, contained any CUGBP1 binding site. Restricting it to binding sites that are 8 nt or longer, only 118 transcripts (6%) were left. Setting the criterion that there have to be at least two distinct binding sites of at least 8 nt length, only 33 transcripts were left (1.7%). Finally, including the criterion that these binding sites have to be at least 100 nt apart to provide some kind of intron in between, only 11 transcripts (0.6%) fulfilled all the criteria. Those 11 transcripts belong to nine different genes (Supplementary Table S5): Superoxide dismutase 2 (SOD2), MAF BZIP Transcription Factor K (MAFK), CUB domain-containing protein 1 (CDCP1), RNA Binding Motif Protein 23 (RBM23), SET Domain Containing Lysine Methyltransferase 7 (SETD7), UBX Domain Protein 2B (UBXN2B), Complexin 2 (CPLX2), CEP41 and UBE2R2. Of these, only CEP41 and UBE2R2 depicted the “decoy regulation pattern.” However, they were not bound by CUGBP1 in the RIP assay of A549 cells (Figure 4C) and therefore excluded as novel decoy targets.

**TABLE 3:** Numbers of transcripts in low stringency analysis.

Binding site length	Number of binding sites	Space between binding sites	Number of transcripts left
–	–	–	575
Binding site of ≥ 8 nt	–	–	118
Binding site of ≥ 8 nt	≥2 binding sites	–	33
Binding site of ≥ 8 nt	≥2 binding sites	≥100 nt apart	11

*For complete list of transcript IDs see Supplementary Table S5. Nt, nucleotides.*

## Discussion

Since their discovery in 1993 (Wightman et al., 1993), miRs were generally considered as gene expression repressors. On post-transcriptional level, miRs specifically bind to 3′UTRs of their target mRNAs which has a silencing effect by either mediating translational repression or degradation of the mRNA (Wienholds and Plasterk, 2005; Croce, 2009). However, over the last years different studies reported that miRs can also positively influence gene expression. They can bind to RNA binding proteins and antagonize their activity which leads to an elevated gene expression of their target genes (Eiring et al., 2010; Saul et al., 2016; Saul et al., 2019a, b). The newly discovered decoy of miR-574-5p and CUGBP1 has a crucial impact on physiological processes, since it regulates mPGES-1, thereby PGE_2_ levels and subsequently lung tumor growth *in vivo* (Saul et al., 2019a). Therefore, it was especially interesting to further characterize this rather unknown miR function which seems to have a more global significance than initially assumed.

In a mass spectrometry-based proteomics study, we aimed to further elucidate global distribution and novel targets of the decoy mechanism. To this end, we conducted a proteomics study with manipulated levels of CUGBP1 or miR-574-5p in A549 lung cancer cells. In this complex study we separately analyzed proteins from soluble and microsomal fraction and observed many compartment specific effects. Especially the R-Smads, SMAD2 and SMAD3 revealed opposite regulation upon ΔCUGBP1 in the two fractions. This supports the hypothesis that RBPs could allow different translational efficiencies concerning free and endoplasmic reticulum-bound ribosomes (Mansfield and Keene, 2009; Reid and Nicchitta, 2012). Moreover, in the two miR conditions (ΔmiR-574-5p and miR574-5p oe), higher percentages of regulated proteins were found in the microsomal fraction, compared to the soluble fraction. This suggests that miR-574-5p is likely to be mainly localized there and further supports the assumption that the decoy mechanism occurs in this fraction. In addition, we also observed that there are more CUGBP1 targets detectable in the microsomal fraction based on the fact that more proteins were upregulated than downregulated upon the knockdown. Whereas, secondary effects were apparently stronger in the soluble fraction, as 8% of all proteins were decreased upon ΔCUGBP1 while only 2% were increased. This also matches the observation that CUGBP1 itself is mainly found in the microsomal fraction.

Hence, we focused on the microsomal fraction in further analyses and identified several new CUGBP1 targets. The NADH-Ubiquinone Oxidoreductase NDUFS2 was already postulated to be directly targeted by CUGBP1 (Rattenbacher et al., 2010). We verified the interaction of CUGBP1 and NDUFS2 via RIP and could confirm the regulation using Western blot analysis. This suggests that CUGBP1 influences the respiratory chain and thereby a major biological process within the cell (Procaccio et al., 1998). Next, we analyzed the SMAD family. SMAD2, SMAD3 and SMAD4 were detected in the proteomics study and expression changes were successfully validated using Western blot analysis. Interestingly, SMAD7, another SMAD family member, was not detected in our proteomics study but nevertheless is described as CUGBP1 target in C2C12, a murine myoblast cell line (Lee et al., 2010). This gives an indication that the SMAD family and subsequent pathways could actually be regulated by CUGBP1 as a regulon. While SMAD2 was significantly enriched in RIPs, SMAD3 showed no enrichment in the CUGBP1-IP. This indicates that SMAD2 is indeed bound by CUGBP1, whereas SMAD3 is not at least under the conditions investigated here. We can probably speculate that the binding of SMAD3 mRNA only takes place under certain conditions, since CUGBP1 is known to bind its targets strictly context specific (Vlasova and Bohjanen, 2008).

Following, we were interested if these new CUGBP1 targets were also influenced by the decoy mechanism. Hence, we took a closer look on their regulation in the microsomal fraction upon miR-574-5p oe and ΔmiR-574-5p. It was expected that targets of the decoy mechanism would be upregulated in response to an overexpression and downregulated in response to a knockdown of the miR-574-5p, as it is the case for mPGES-1. However, microsomal SMAD2 and NDUFS2 did not exhibit this “decoy regulation pattern” which implies that they are not affected by the decoy mechanism. In fact, the only protein depicting the “decoy regulation pattern” in the Western blot analyses was mPGES-1. However, it should be mentioned that mPGES-1 did not reveal this pattern in the proteomics data. We would like to point out that a proteomics study, even if we have used an established method (Eriksson et al., 2008; Ochs et al., 2013; Bergqvist et al., 2019; Saul et al., 2019b), only provides initial indications of which proteins could be regulated. It does not map the exact expression changes, which is a common limitation of this approach. It is therefore possible that mPGES-1 as multi-pass membrane protein is not efficiently, quantitatively represented by the mass spectrometry due to different sample preparation procedures. In contrast to mass spectrometry samples, protein samples in Western blot analyses are SDS-treated and boiled. Therefore, it is likely that Western blot analysis allows for a better quantification in these cases. This also means, it is possible that there are novel decoy targets which we did not detect with our proteomics approach. To ensure the general accuracy of the study, it is crucial to validate the mass spectrometry data. We addressed this for a selected amount of proteins and further supported this by additional validation of the pathway analysis which is in turn based on the proteomics study.

Interestingly, the decoy mechanism of miR-328 and hnRNP E2 affects a variety of targets and cellular processes (Saul et al., 2016, 2019b) while in this case the decoy of miR-574-5p and CUGBP1 seems to be quite specific for mPGES-1. Retrospectively, this is consistent with one of our previous studies in which we firstly demonstrated that miR-574-5p prevents CUGBP1 from binding to mPGES-1 mRNA. This has a crucial impact on lung tumor growth *in vivo* (Saul et al., 2019a) due to the influence on PGE_2_ levels and the tumor microenvironment. However, those pro-tumorigenic effects are completely blocked with the administration of a selective mPGES-1 inhibitor and apparently are solely caused by the decoy-mediated mPGES-1 induction. Therefore, it seems likely that the decoy function mainly regulates mPGES-1 expression which might be a cell-type-specific effect.

The hypothesis is further supported by the fact that CUGBP1 and miR-574-5p are mainly located in the nucleus. It is implied that the role of CUGBP1 as regulator of alternative splicing is of greater importance here than its function as translational repressor (Dasgupta and Ladd, 2012). Therefore, it was assumed that miR-574-5p could solely interfere with this CUGBP1 function. Thus, we analyzed the splicing pattern that emerged from the only known decoy target mPGES-1. It has a specific pattern comprising of two, 39 and 46 nt long CUGBP1 binding sites, separated by a 1000 nt 3′UTR intron (Saul et al., 2019a). Alternative 3′UTR splicing leads to the generation of a mPGES-1 isoform with a much higher translational rate. This can be explained by the fact that inhibitory elements like Alu elements or canonical miR binding sites within the 3′UTR intron are removed (Daskalova et al., 2007; Mayr and Bartel, 2009; Fitzpatrick and Huang, 2012). In a high stringency analysis, we discovered that this specific splicing pattern is exclusively found for mPGES-1 and could not be observed for another potential CUGBP1 target. This can be explained by the fact that this splice pattern with a 3’UTR Alu element framed by two CUGBP1 sites is indeed very specific. Thus, we decided to loosen the criteria and looked for similar patterns in a second low stringency analysis which resulted in a list of 11 transcripts. Taking into account that we started with nearly 2000 transcripts, this gave us another strong hint that the decoy mechanism seems to be very specific. In the future it would be necessary to examine these transcripts more closely for example in relation to splice variants under inflammatory or pro-tumorigenic conditions. Further, it should be noted that we analyzed the 3′UTRs of the transcripts, as we used mPGES-1 as model for a decoy target. Despite the fact that CUGBP1 indeed mostly binds to 3′UTRs of target genes, it is also known to bind to exon-intron boundaries within the coding sequence (Xia et al., 2017) which we did not include within this study.

Taken together, in this study we discovered a variety of potential new canonical targets of CUGBP1 and successfully verified two of them. By influencing NDUFS2 expression, CUGBP1 is able to interfere with the respiratory chain and could have an impact on the mitochondrial complex I (Procaccio et al., 1998). The newly found interaction with SMAD2 mRNA opens further possibilities for CUGBP1, since SMAD2 mediates transforming growth factor β signaling and has a crucial impact on many signaling cascades (Riggins et al., 1996).

Unexpectedly, we found that the decoy mechanism of CUGBP1 and miR-574-5p seems to be quite target-specific in this type of lung cancer cells. Overall, we used several criteria to identify new targets of the CUGBP1/miR-574-5p decoy mechanism: First, potential candidates should show a “decoy regulation pattern” on protein level as we have seen for mPGES-1. Second, the splice site in the 3′UTR should contain two CUGBP1 binding sites with a potential intron in between. Third, binding of CUGBP1 to the respective binding sites occurs. Interestingly, only mPGES-1 was identified which fulfills all of these three criteria (Figure 5C). Our data suggest that decoy mechanisms can lead to the regulation of specific target genes which is potentially cell-type-specific. Interestingly, in A549 cells, mPGES-1 was identified as the only protein regulated by the decoy mechanism of CUGBP1 and miR-574-5p. This explains our previous observation that tumor growth induced by overexpression of miR-574-5p can be selectively blocked with an mPGES-1 inhibitor (Saul et al., 2019a). Concerning lung cancer, mPGES-1 could be a promising target for future lung cancer therapy in patients overexpressing miR-574-5p.

In light of the observation that not all patients benefit from a treatment with PGE_2_-reducing medication (Edelman et al., 2015; Yokouchi and Kanazawa, 2015), determination of miR-574-5p levels might be helpful to identify PGE_2_-dependent tumors. Hence, the levels of miR-574-5p could serve as stratification marker to identify subgroups of patients for the treatment with mPGES-1 inhibitors.

## Data Availability Statement

The mass spectrometry proteomics data have been deposited to the ProteomeXchange Consortium via the PRIDE (Perez-Riverol et al., 2019) partner repository with the dataset identifier PXD016803.

## Author Contributions

AE performed the experiments, analyzed the data, and wrote the manuscript. JW performed the immunostaining and FISH experiments. EO, IB, JL, and KK performed the MS analytic. P-JJ and DS conceived the study and designed the project. MS conceived the study, designed and supervised the project, and wrote the manuscript. All authors conducted the quality assurance of the manuscript and reviewed the manuscript.

## Conflict of Interest

The authors declare that the research was conducted in the absence of any commercial or financial relationships that could be construed as a potential conflict of interest.

## Footnotes

1 www.ingenuity.com

2 https://genome.ucsc.edu/

## Abbreviations

Δ: knockdown
CUGBP1: CUG-RNA binding protein 1
FISH: fluorescence *in situ* hybridization
hnRNP E2: Heterogenous Nuclear Ribonucleoprotein E2
IL: Interleukin
IPA: Ingenuity pathway analysis
miR: microRNA
mPGES-1: microsomal prostaglandin E-synthase-1
NDUFS2: NADH- Ubiquinone Oxidoreductase Core Subunit S2
nt: nucleotides
oe: overexpression
PGE_2_: prostaglandin E_2_
RBP: RNA-binding protein
RIP: RNA immunoprecipitation
SEM: standard error of mean
SMAD: Mothers against decapentaplegic homolog
TMT: tandem mass tag
UTR: untranslated region.
