# Supplementary material for: Proteomics-Based Characterization of miR-574-5p Decoy to CUGBP1 Suggests Specificity for mPGES-1 Regulation in Human Lung Cancer Cells
Source: Front Pharmacol. 2020 Mar 13;11:196. doi: 10.3389/fphar.2020.00196 (PMC7082395; doi:10.3389/fphar.2020.00196)
Supplement: Supplementary file 1 [file Data_Sheet_1.PDF]

Supplementary Material

Proteomics-based characterization of miR-574-5p decoy to CUGBP1 suggests specificity for mPGES-1 regulation in human lung cancer cells

Anne C. Emmerich [1,2], Julia Wellstein [1,2], Elena Ossipova [3], Isabell Baumann [1,2], Johan Lengqivst [3], Kim Kultima [4], Per-Johan Jakobsson [3], Dieter Steinhilber [2], Meike J. Saul [1,2,*]

[1] Department of Biology, Technische Universität Darmstadt, 64287 Darmstadt, Germany

[2] Institute of Pharmaceutical Chemistry, Goethe Universität Frankfurt, 60438 Frankfurt/M., Germany

[3] Rheumatology Unit, Department of Medicine, Solna, Karolinska Institutet, Karolinska University Hospital Solna, SE- 17176 Stockholm, Sweden

[4] Department of Medical Sciences, Clinical Chemistry, Uppsala University, SE- 751 85 Uppsala, Sweden

*** Correspondence:**
Dr. Meike J. Saul

saul@bio.tu-darmstadt.de

1 Supplementary Data

Table S1: TMT proteomics data soluble fraction. (file type: xlsx (Microsoft Excel file))

Table S2: TMT proteomics data microsomal fraction. (file type: xlsx (Microsoft Excel file))

Table S3: Upstream regulators predicted by Ingenuity pathway analysis. (file type: xlsx (Microsoft Excel file))

Table S4: Top canonical pathways predicted by Ingenuity pathway analysis. (file type: xlsx (Microsoft Excel file))

Table S5: Transcript List of low stringency 3'UTR analysis (file type: xlsx (Microsoft Excel file))

Figure S1: Transfection efficiency of miR-574-5p overexpression and knockdown of miR-574-5p and CUGBP1 (file type: tif)

Figure S2: Proteomics validation of NDUFS2, SMAD2, SMAD3 and mPGES-1 upon ΔCUGBP1, ΔmiR-574-5p or miR-574-5p oe in IL-1β stimulated A549 cells in soluble and microsomal fraction. (file type: tif)

Figure S3: Further proteomics validation of SMAD4 and p38 upon ΔCUGBP1 in IL-1β stimulated A549 cells in soluble fraction. (file type: tif)

2 Supplementary Figures and Tables

2.1 Supplementary Figures

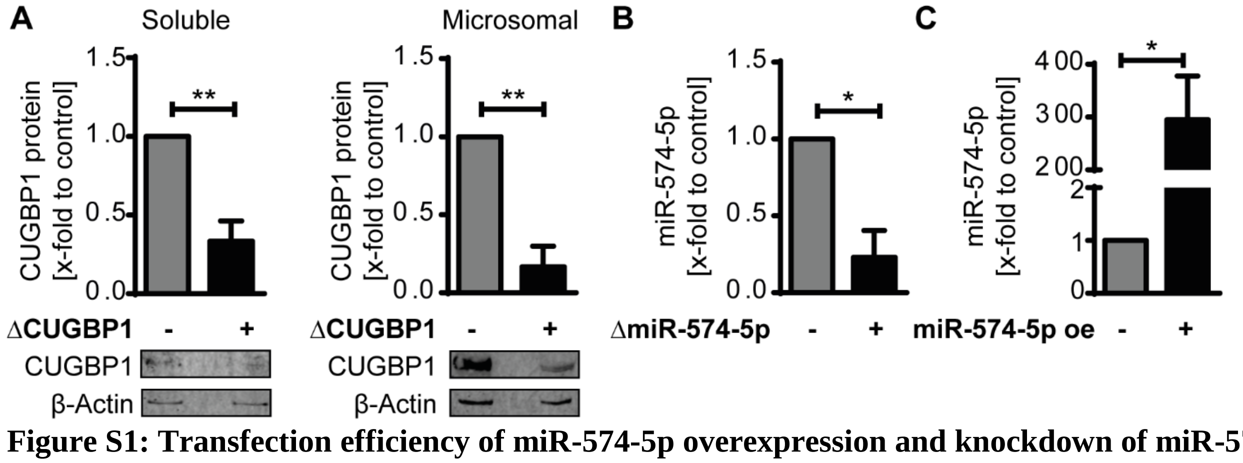

Figure S1: Transfection efficiency of miR-574-5p overexpression and knockdown of miR-574-5p and CUGBP1. (A) CUGBP1 was depleted by 67% in soluble and 83% in microsomal fraction. β-Actin served as loading control. (B) miR-574-5p was reduced by 77%, while (C) significant oe was 294-fold, both quantified using qRT-PCR. Protein an miR levels were compared to each control (set as 1) and relative changes are given as mean (+SEM) of three independent experiments, *p≤0.05, **p≤0.01. Δ: knockdown, oe: overexpression. (fily type: tif)

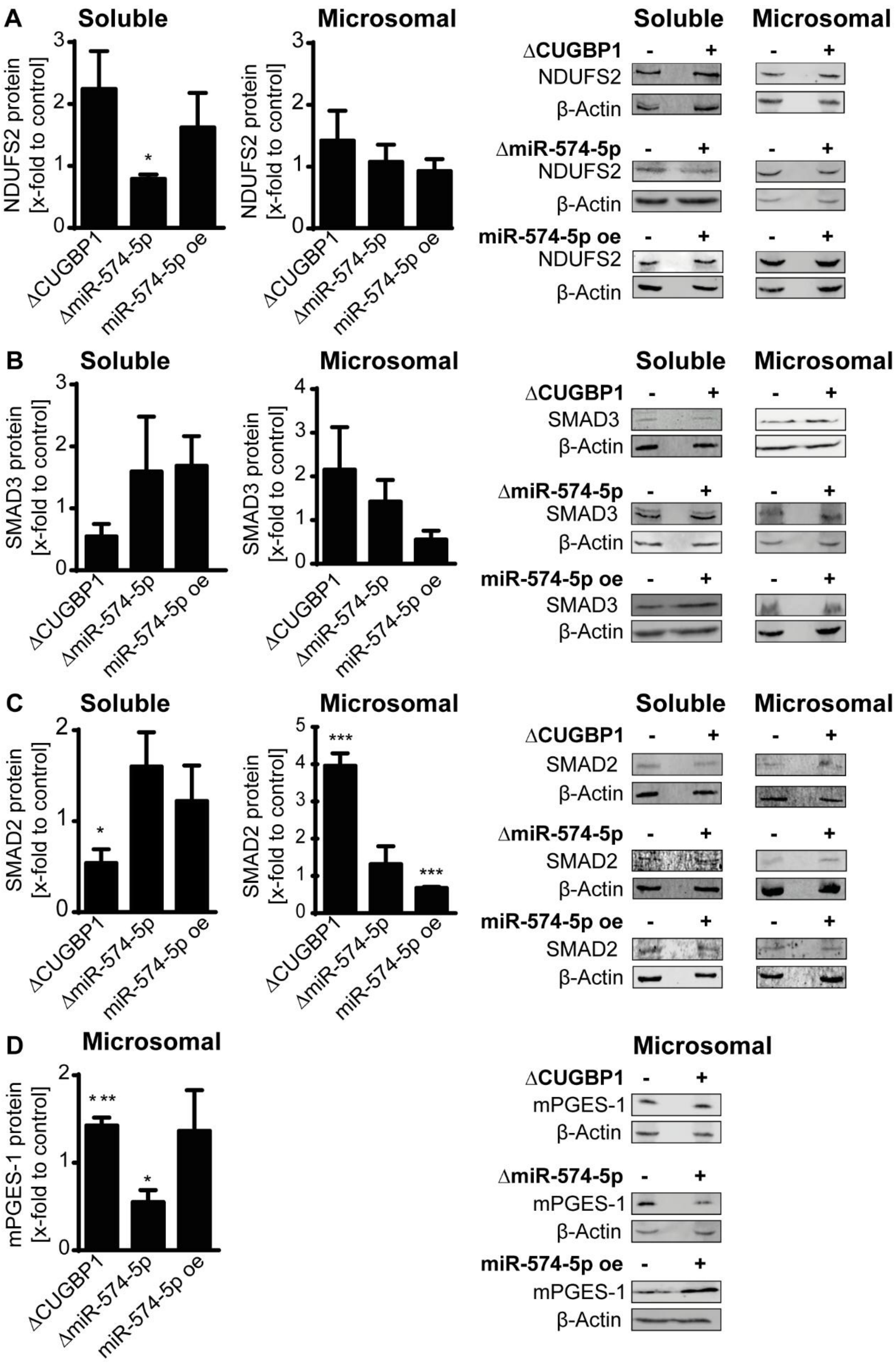
A
Soluble
Microsomal
NDUFS2 protein [x-fold to control]
ΔCUGBP1
ΔmiR-574-5p
miR-574-5p oe
Soluble
Microsomal
ΔCUGBP1 - +
NDUFS2
β-Actin
ΔmiR-574-5p - +
NDUFS2
β-Actin
miR-574-5p oe - +
NDUFS2
β-Actin
B
Soluble
Microsomal
SMAD3 protein [x-fold to control]
ΔCUGBP1
ΔmiR-574-5p
miR-574-5p oe
SMAD3
β-Actin
C
Soluble
Microsomal
SMAD2 protein [x-fold to control]
ΔCUGBP1
ΔmiR-574-5p
miR-574-5p oe
SMAD2
β-Actin
D
Microsomal
mPGES-1 protein [x-fold to control]
ΔCUGBP1
ΔmiR-574-5p
miR-574-5p oe
mPGES-1
β-Actin

Figure S2: Proteomics validation of NDUFS2, SMAD2, SMAD3 and mPGES-1 upon ΔCUGBP1, ΔmiR-574-5p or miR-574-5p oe in IL-1β stimulated A549 cells in soluble and microsomal fraction. Validation of proteomics data using Western Blot analysis of (A) NDUFS2, (B) SMAD3, (C) SMAD2 and (D) mPGES-1 in IL-1β-stimulated A549 cells upon ΔCUGBP1, ΔmiR-574-5p or miR-574-5p oe. β-Actin served as loading control. Protein levels were compared to each control (set as 1) and relative changes are given as mean (+SEM) of three to six independent experiments, *p≤0.05, ***p≤0.001. Δ: knockdown, oe: overexpression (file type: tif)

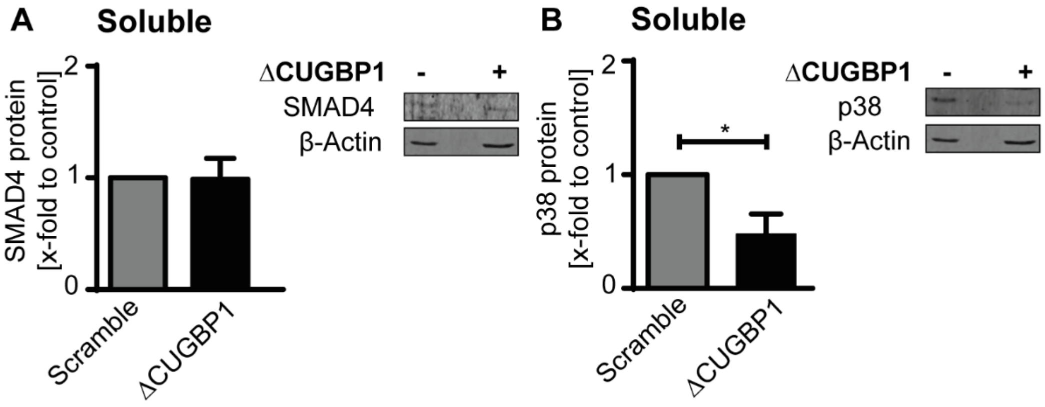

Figure S3: Further proteomics validation. Levels of SMAD4 and p38 upon ΔCUGBP1 in IL-1β stimulated A549 cells in soluble fraction. Validation of proteomics data using Western Blot analysis of (A) SMAD4, (B) p38 in IL-1β-stimulated A549 cells upon ΔCUGBP1. β-Actin served as loading control. Protein levels were compared to scramble control (set as 1) and relative changes are given as mean (+SEM) of three independent experiments, *p≤0.05. Δ: knockdown, oe: overexpression (file type: tif)
